# Extreme heat and cultural and linguistic minorities in Australia: perceptions of stakeholders

**DOI:** 10.1186/1471-2458-14-550

**Published:** 2014-06-03

**Authors:** Alana Hansen, Monika Nitschke, Arthur Saniotis, Jill Benson, Yan Tan, Val Smyth, Leigh Wilson, Gil-Soo Han, Lillian Mwanri, Peng Bi

**Affiliations:** 1 Discipline of Public Health, The University of Adelaide, Level 8, Hughes Building, Mail Drop DX650 207, Adelaide, South Australia 5005, Australia; 2 Department for Health and Ageing, Adelaide, SA, Australia; 3 Division of General Practice, The University of Adelaide, Adelaide, SA, Australia; 4 Discipline of Geography, Environment and Population, The University of Adelaide, Adelaide, SA, Australia; 5 School of Science and Health, University of Western Sydney, Sydney, NSW, Australia; 6 Communications and Media Studies, Monash University, Clayton, VIC, Australia; 7 Faculty of Nursing, Medicine and Health Sciences, Discipline of Public Health, School of Health Sciences, Flinders University, Bedford Park, SA, Australia

**Keywords:** Extreme heat, Climate change, Migrants, Australia

## Abstract

**Background:**

Despite acclimatisation to hot weather, many individuals in Australia are adversely affected by extreme heat each summer, placing added pressure on the health sector. In terms of public health, it is therefore important to identify vulnerable groups, particularly in the face of a warming climate. International evidence points to a disparity in heat-susceptibility in certain minority groups, although it is unknown if this occurs in Australia. With cultural diversity increasing, the aim of this study was to explore how migrants from different cultural backgrounds and climate experiences manage periods of extreme heat in Australia.

**Methods:**

A qualitative study was undertaken across three Australian cities, involving interviews and focus groups with key informants including stakeholders involved in multicultural service provision and community members. Thematic analysis and a framework approach were used to analyse the data.

**Results:**

Whilst migrants and refugees generally adapt well upon resettlement, there are sociocultural barriers encountered by some that hinder environmental adaptation to periods of extreme heat in Australia. These barriers include socioeconomic disadvantage and poor housing, language barriers to the access of information, isolation, health issues, cultural factors and lack of acclimatisation. Most often mentioned as being at risk were new arrivals, people in new and emerging communities, and older migrants.

**Conclusions:**

With increasing diversity within populations, it is important that the health sector is aware that during periods of extreme heat there may be disparities in the adaptive capacity of minority groups, underpinned by sociocultural and language-based vulnerabilities in migrants and refugees. These factors need to be considered by policymakers when formulating and disseminating heat health strategies.

## Background

Each summer Australia experiences periods of very hot temperatures, and extended heatwaves with maximum temperatures exceeding 35°C for several consecutive days are not uncommon. Despite the population being acclimatised, thermal tolerance can be exceeded when heat extremes occur, and the consequent health impacts can range from marginal increases in morbidity to significant increases in mortality [1-4]. It is well established that the elderly, the young and the sick are disproportionately at risk [3]. However, other subgroups are also vulnerable and with warmer temperatures imminent, identifying these groups is important for public health authorities in formulating targeted inventions.

The number of permanent immigrants to Australia has increased over several decades to the extent that more than one quarter of the nation’s population of 23.3 million is overseas-born, and a further one fifth has at least one parent born overseas. Immigrants include those arriving through the Migration Program (including skilled and family stream migrants) and the Humanitarian Program for refugees forced to leave their homeland [5]. Many migrants arrived from South-East Asia in the 1970s and in recent years the proportion of migrants from Asia, as well as other countries, has increased. According to the 2011 national census, almost half of the ‘long-standing migrants’ who arrived before 2007 and more than two thirds of the recently arrived, speak languages other than English at home [6].

The impacts of heat on the health of people in migrant and minority groups are not well documented. Some studies conducted in the United States have shown that heat-related deaths can be high in people of African American descent [7-10], and undocumented immigrants from Mexico entering the United States across borders adjoining the Arizona desert [11,12]. However, in countries outside of the United States the literature on susceptibility and adaptation to heat in culturally diverse groups is scarce. This may be due to the issue not previously being seen as an area of public health concern or that data have not been readily available for epidemiological studies. Ancestry has not been well recorded in health statistics to date, and people in non-English speaking communities have often been excluded from traditional health research studies [13].

Despite the nation’s diversity in cultures, language and climate experiences, it is unknown if migrants seamlessly adapt to Australia’s hot summers or if certain barriers are encountered which could affect wellbeing during bouts of extreme heat. This is a critical gap in public health knowledge, particularly in countries where migration and cultural diversity is increasing and the climate is warming. The aim of this research was to ascertain using qualitative methods, if barriers affecting adaptation to extreme heat exist within culturally and linguistically diverse (CALD) communities in Australia and if so, to identify vulnerable subgroups.

## Methods

**Figure 1:**
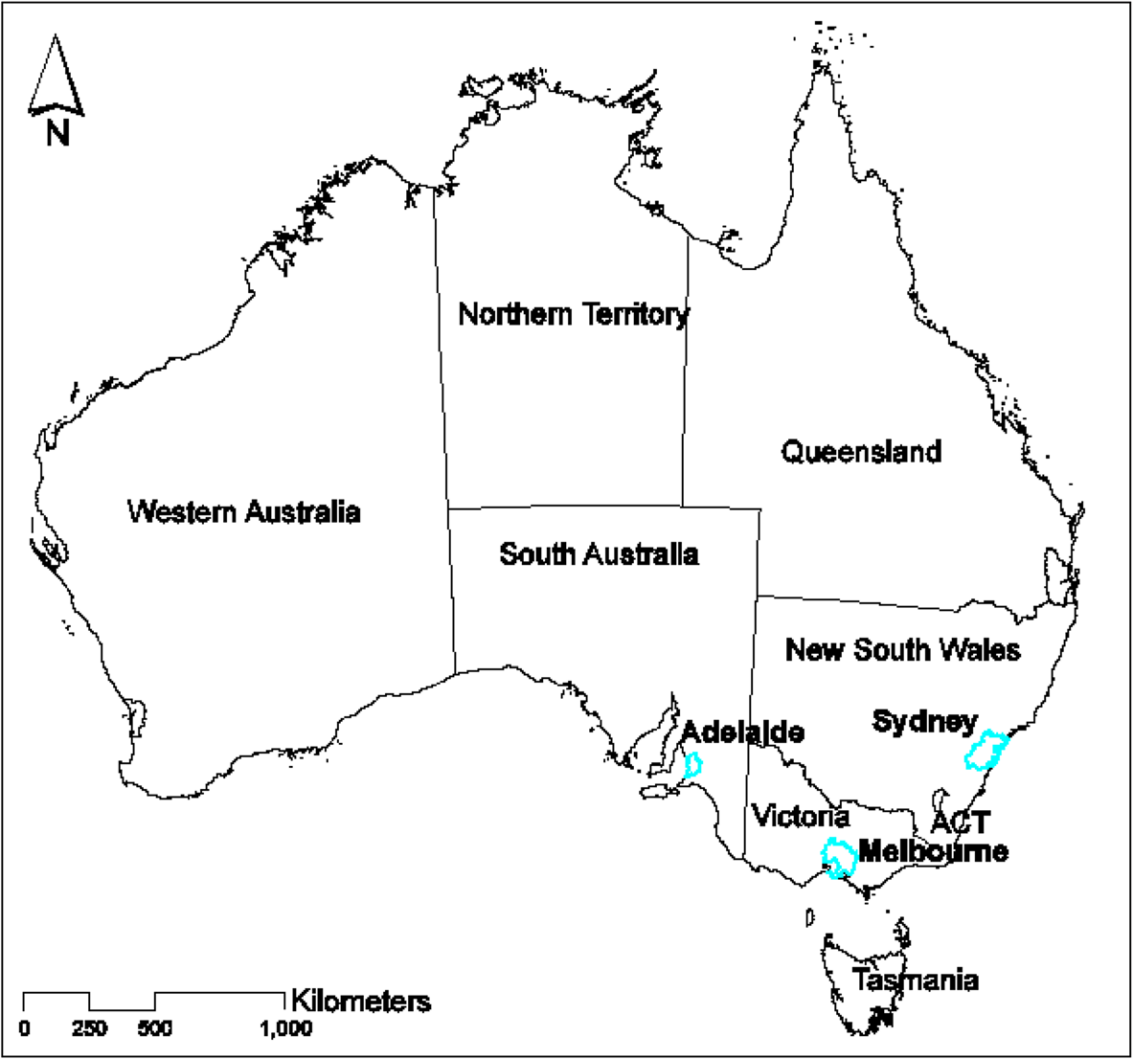
Map showing study locations of Adelaide, Melbourne and Sydney, Australia.

The study was based in Adelaide, South Australia, and data were also collected in Melbourne, Victoria; and Sydney, New South Wales (Figure 1). Of the three cities, Adelaide has the lowest population (1.2 million) and the warmest climate; Melbourne has a population of 4.1 million and the coolest climate; and Sydney has the largest population (4.6 million) and a more humid climate [5]. The percentage of residents born overseas in the respective states of South Australia, Victoria and New South Wales is 22%, 26% and 27% respectively [5].

Cross-cultural research calls for a degree of flexibility in sampling and recruitment as standard community sampling techniques can be unduly time-consuming and expensive [14]. Careful consideration was therefore given to a sampling strategy that would adequately answer the research question whilst providing information about issues affecting a range of immigrants of diverse cultural and linguistic backgrounds, ages, and length of stay in Australia. Hence, using purposive sampling methods, key informants closely associated with a range of migrant groups were identified through a research reference group and a comprehensive internet search of services and support groups. Stakeholders from three main sectors (state and local government; non-government organisations and service providers; and migrant and refugee health services) were contacted by telephone and/or email and interested persons provided with further information. On occasions the primary contact declined to participate and suggested a secondary contact more experienced in the research topic. An advantage of our sampling strategy was that stakeholders acted as conduits and were able to speak freely about their observations and experiences of barriers and enablers encountered by clients and community members. Snowball sampling also resulted in a convenience sample of members of an Asian community group and a recently arrived refugee family.

Interviews and group interviews/focus groups (with between two and five participants) were conducted in the warm months between December 2011 and April 2012 in a range of venues. In Adelaide, interviews and focus groups were held at the University of Adelaide or on site at the respondents’ organisation. In Melbourne, sessions were held at the respondents’ place of employment, and a family home, whilst in Sydney sessions took place at venues in the multicultural inner west/western suburbs. Informed, written consent was provided by respondents prior to the commencement of the interviews and confidentiality was assured. For one community group information sheets and consent forms were translated and a bi-lingual speaker assisted with the session.

The interview topic guide was informed by a literature review and the research reference group comprising a panel of experts. Questions related to experiences with extreme heat in the communities, factors contributing to vulnerability, adaptive behaviours, and knowledge of heat-health warnings, as detailed previously [15]. Whilst the questions for community members were essentially the same as those for other stakeholders, the wording was modified slightly. Respondents were encouraged to use the questions as a guide only and to expand on points of interest. All except one interview was digitally recorded and subsequently transcribed by either the researcher or an independent service.

### Data analysis

Transcripts were imported into the qualitative data analysis software package NVivo 9 (QSR International, Doncaster, Australia). Data for each city were analysed separately using the framework approach. Described by Ritchie and Spencer (1994), framework analysis uses a systematic approach to data management to provide coherence and structure to qualitative data [16,17]. Passages of text representing repeated themes were identified and assigned headings according to the context and coded to as many relevant categories as possible to reduce the likelihood of missing key points. The data were then synthesised in a chart format using headings identified from the thematic analysis [16]. This approach enhances rigour, transparency and validity of the analytic process [18]. Analysis was both deductive, with categories derived from prior knowledge, and inductive, with categories emerging purely from the data [19].

Ethics approval for the study was received from the University of Adelaide, Monash University and the South Australian Department of Health. The study adheres to the ‘RATS’ qualitative research review guidelines for reporting qualitative studies (http://www.biomedcentral.com/authors/rats) (Additional file 1).

## Results

In total there were 36 respondents across the three cities, with the majority being from Adelaide (Table 1). Most were involved in service provision to, and liaison with, clients in CALD communities. Many were migrants themselves (or descendants of immigrants) from Africa, Asia, Europe or the Middle East.

**Table 1:** Numbers of respondents in each city and affiliations

**Sector**	**Adelaide**	**Melbourne**	**Sydney**
State and local government	7	2	4
NGO	10	1	2
Health sector	4	0	1
Community members	0	3	2
**TOTAL**	**21**	**6**	**9**

Respondents spoke about the barriers and enablers facing some in migrant and refugee communities during periods of extreme heat in Australia, and also commented that some were relatively unaffected. In depth narratives revealed the disparities between the communities regarding abilities to cope with heat and one respondent spoke of the migrant population not being considered by authorities:

“Extreme heat, it happens every year but nobody thinks of the migrants and how it affects them, … OK, you survive like everybody else but not everybody is prepared the same way for it and not everybody has the resources to manage that time.”

Coordinator, Adelaide

Eleven emergent and often inter-linking themes were identified from the narratives: ‘Cultural factors’, ‘Fluid intake’, ‘Health issues’, ‘Heat is different’, ‘Housing’, ‘Language barriers’, ‘Isolation’, ‘Low literacy’, ‘Power costs’, ‘SES’, and ‘Transport’. Displayed in Table 2 are the themes from the Adelaide narratives, some of which were reiterated interstate - i.e. the first six of these eleven themes also emerged from the Melbourne narratives, whilst from Sydney there were five (‘Cultural factors’, ‘Health issues’, ‘Housing’, ‘Language barriers’ and ‘Power costs’). Another theme identified in each city was ‘Who is vulnerable?’

**Table 2:** Vulnerability factors identified from Adelaide data

**No.**	**Cultural factors**	**Fluid intake**	**Health issues**	**Heat is different**		**Housing**
1	Not culturally acceptable for some people to pick up information pamphlets which need to be given.	Messages should specify that people need to drink water. Incontinence issues can be a problem in older people.	Many in new & emerging communities have high blood pressure, mental health problems including grief and loss issues, kidney problems, and nutritional deficiencies. Health issues with older people.	Climate is different (very dry) here. Cannot assume people from hot areas will be OK in a heatwave.		Many have adapted their houses to suit the climate. Less so for those that rent. Fear can prevent people opening their house at night. No air conditioning (AC) supplied in public housing.
2	Some women in new communities are illiterate. Concern for veiled women in the heat.	Middle Eastern men often don’t drink enough water.	Vitamin D deficiency common in Middle Eastern females, kidney stones in males. People fearful of melanoma.	The heat is very different and dry. People not told about the heat. Assumptions can be made about people’s heat tolerance.		Mothers on Women at Risk visas in low quality rental housing - pull down blinds, open up house, use fans.
3	Older African people are unable to keep cool in traditional ways. The children are unable to speak the dialects of the older people. Education needed in the communities about dressing for hot weather	Cold water can be unpalatable to some.	Being confined to the home during the heat can be emotionally disturbing. Some believe it is unhealthy to have a fan blowing directly on them.	Not as hot in Africa as in Australia. People who work on farms in Africa jump in streams to cool down. Here they sit inside the house.		
4	Some cultures prefer hot food to cold; but cooking heats up houses. Food spoilage can be an issue. Asian women use sun umbrellas. In older persons first culture reversion can lead to overdressing.	In some cultures drinking water is more acceptable than in others.	Some people will not use air conditioning if they are not feeling well.	Different climate pattern in Australia. Drier heat which may not cool down in evening. Assumption that people from hot climates can adapt. Sunburn can be a problem.		Past issues with new arrivals not knowing how to use household appliances.
5	Many are wary of local government. Church representatives may be able to relay heat messages.		Older people often think they will cope like a younger person. Visual impairments in the aged can lead to air conditioners being set to ‘heat’ not ‘cool’.			Some Italian and Greek migrants have lush gardens and significant trees that shade houses.
6	Ramadan can be an issue if it falls in hot weather. Cultural sensitivity require the wearing of cultural garments in the heat. Education needed re dressing children for hot weather. Dark clothing can be worn during periods of grieving.	Some people prefer to drink hot rather than cold water.	Some shopping centres have removed seating. Access to air conditioning can save lives but some won’t use it.	In Australia it is different to Africa where it is tropical, with a cool wind. The newly arrived don’t know how hot it gets. Heat is dry here.		Many live in homes without AC or in old homes that take days to cool down. In first 6 months accommodation is basic, crowded. Asylum seekers may have no secure housing.
7	Electrical appliances can be new to refugees. Swimming often not part of cultural norm. People socialise less with neighbours here. Cultural issues with fluid intake and Ramadan, and cultural dress codes during hot weather.	Some in new communities get dehydrated - need to be reminded to drink water. Some don’t like the taste. Water required boiling in refugee camps.	Sun here burns the skin. Gallstones common as people don’t drink enough. There can be religious and cultural barriers for CALD women seeking health care and treatment in emergency departments.	Different type of heat and it doesn’t cool down at night. The heat is very direct, dry, and uncomfortable. Incorrect assumptions that people from hot countries are used to the heat.		Houses may not be in good condition. There should be fans in each bedroom. If their house is hot people will go to shopping centres.
8	People can feel a responsibility to financially support family in their home country. Some with distinguishing skin colour feel as if they stand out in shopping centres. People may not intuitively know how to dress for the heat.	Many don’t find cold water palatable.	Having doors open can let in hot air and be dangerous. Those without AC at home or in the car can find it very tormenting.	Hotter, drier than in Africa. Even the breeze is hot.		Some refugees buy fans but have windows and doors open so just hot air blowing. Houses often have no AC.
14	Information in two dimensional formats can be new to some without literacy skills.		Cognitive impairments and chronic conditions add to vulnerability. People can be unaware of the impact heat and dehydration can have on the elderly. Electricity concessions are available to some.			Some homes are old, no AC, no insulation. People spend most of their time in the house. Focus should be on improving housing for the most vulnerable with the least income.
**No.**	**Isolation**	**Language barrier**	**Low literacy**	**Power costs**	**SES**	**Transport**
1	Ethnicity can add to feeling of isolation in older people. Some have no family, don’t know their neighbours and have no support network. Difficult to get information to the isolated.	Language a barrier in new communities. Not only the language but the context can be a problem – the term ‘heatwave’ may not be understood.	Low literacy levels in some CALD populations. Many obstacles to getting literature translated.	People won’t put AC on if they can’t afford it no matter what their nationality. Older people won’t use AC due to cost.	If people are of low socio-economic status (SES) e.g. refugees, they won’t use AC. Older migrants are frugal.	Not all councils have community transport to get people to community centres. Messages could be disseminated via TV on buses.
2	Clients are well connected with their extended families so isolation should not be a problem.	Some young female Liberian immigrants are not literate in their language.	Young Liberian mothers can have poor literacy and can’t access warnings. African culture can be verbal-oral rather than literal.	Young refugee mothers with babies won’t use AC due to the cost of electricity.	Low SES people can’t afford the cost of electricity	Most refugees don’t drive. Have to use public transport and stand at bus stops without shelters in the heat.
3	Confinement in the house when it is hot can be emotionally disturbing.	Much harder for older people to learn new language. African children may not speak the dialects of the older people.		People don’t want to use AC because of the financial aspect	People can’t afford to register at a swimming pool. Financial barriers in low SES families.	Refugees may not drive or have vehicles. Difficult for older ones to learn to drive.
4		First language reversion common in the elderly due to age, dementia, stroke. Lesser need for migrants to use English as they age.		People in new communities may not be familiar with AC and can’t afford power costs.	More money equals better rights to information and more choices.	
5	Older people can be socially isolated. Less isolation in extended families.	Having poor English is a huge barrier. Elderly people can revert to their first language. Information needed in other languages.	People might not be literate in English or their own language. Many older migrants had low literacy skills when they emigrated.	The increasing cost of power is a huge issue for older people. They won’t use AC because of stress about getting the bill.	Older people can be asset rich, cash poor so will not get new AC or get the old one fixed	Older people can be isolated and unable to access transport
6	Some have no family and friends. Service providers may be their only contact. Asylum seekers may not be connected at all. New arrivals may not know who to contact for help.	Language specific radio stations are popular with older migrants.		The elderly won’t turn on AC because of the cost. They will put up with a few days of heat rather than waste the money.	SES a key factor. New arrivals have basic accommodation at first. Asylum seekers may have no income, Medicare, accommodation, phone or TV.	Caution required when recommending older people leave home in the heat if they have to catch a bus. Council’s community buses are useful.
7	Neighbours don’t socialise as much in Australia.	Information not in own language can’t be understood. Lack of interpreter can be a barrier to health care.	Some migrants have never been to school and can’t read information pamphlets	Many are concerned with power costs and won’t use fans.		Difficulties using public transport with babies and prams. Some bus drivers are unhelpful.
8		People won’t get messages if they’re in English, but not practical to have in all dialects. Need information at orientation instead.		Low income refugees can’t afford to run AC. They will open windows instead.	Refugees go without so they can send money to family back home. Strong sense of responsibility to help relatives.	
14	Those with no family connection are isolated and vulnerable. People can be invisible in the community. Non-English speaking clients can’t access services in English.	Translating written material is ineffective. Phone contact best. Interaction the key. First language reversion occurs with dementia.	Translating is a problem as some people are illiterate in their own language. Some post WWII migrants had little schooling.	The cost of electricity is a barrier for clients	Financial assistance required for some to install AC. Most vulnerable with least income have inefficient heating, cooling.	Transport for clients cancelled when hot for health reasons. People are advised to go to cooler places but the elderly often cannot.

### Cultural factors and norms

Although many new arrivals adapt quickly in Australia, some can be unaware of the need for adults and children to dress lightly during the heat to aid thermoregulation. Additionally, some cultural and religious mores at times dictate the wearing of traditional heavy, dark coloured garments not ideally suited to hot weather.

A culture-specific barrier that was raised in Melbourne and Adelaide by stakeholders from Africa was that sometimes people in visible minorities reportedly do not feel comfortable “*hanging around*” in cooled spaces such as shopping centres because “*you stand out when you’re different*”. By contrast, a refugee from Bhutan stated that going to shopping centres was a more practical alternative for his community than cooling off at swimming pools, because “*95% of Bhutanese they don't know how to swim”.*

Cultural differences surrounding preferences for hot food and being unable to drink between dawn and sunset during the Islamic month of fasting (Ramadan) can be problematic during hot weather. Respondents also highlighted that due to previous experience in their home countries, many migrants are wary of officials or people in uniform offering assistance, and that access to culturally appropriate emergency health care can be an issue for some women.

The practice of Asian women using sun umbrellas to preserve skin colour and Muslim women wearing culturally appropriate swimwear were mentioned as examples of cultural adaptation to the hot climate. The strong family connections and social networks of migrant groups can be beneficial during the heat, particularly for older people in CALD communities who are cared for by their families. By contrast, a Sydney respondent mentioned that the cultural norm of elders living with their family may not always reduce vulnerability if there is a disincentive for them to use air conditioning because of the added cost to the household.

### Health issues and lack of fluid intake

Some migrants and refugees do not drink enough water for reasons which include a dislike of the taste, a lack of awareness about the need to keep hydrated during hot weather and recollection of poor water quality in refugee camps. However, one respondent stated he drank more water now than when he was in Africa. Another spoke of people who have built up a “*resistance*” to lack of water because of past experiences, and can “*go for hours without water*”. Health care providers and others also spoke of people having insufficient fluid intake leading to health issues such as kidney stones, gall stones, headaches and constipation. A manager spoke about refugees preferring soft drinks to water as it is a “*sign of affluence*” and of the consequent impact on physical and dental health. Another respondent pointed out that promoting water to migrants as the “*standard drink*” should be encouraged. It was mentioned that for older people, a reluctance to drink water can be related to incontinence issues and that messages about dehydration for the young as well as older people, need to be reinforced:

“But I still think a lot of the key messages in keeping hydrated and what to do when working with young children or caring for young children, some of those messages I don’t think are still reaching the communities.”

Diversity Officer, Melbourne

A physician in Adelaide mentioned that people in new and emerging communities can have a range of co-morbidities, nutritional deficiencies and mental health issues which can affect vulnerability. Also mentioned was the mental anguish that can be experienced during periods of extreme heat by being confined to a hot house. Strong descriptive terms such as “*emotionally disturbing*” and “*tormenting*” were used.

Health issues were raised by respondents in Melbourne who spoke about the effect of the dry heat causing people to “*feel exhausted and tired*” and that chronic health conditions influenced vulnerability. Valuable information about heat and its effect on the health of new arrivals was gained from discussion with a refugee family. When asked if very hot weather affects how people feel, the respondent answered passionately that it was “*affecting the total health of the people*”. He spoke about headaches, feeling lazy, itchy skin rashes and sunburn. The respondent expanded on the lack of acclimatisation and underlying health problems that could be contributing factors:

“They came from refugee background so they never had proper amount of nutrition food in their camp life and lack of light so they lack vitamin D as we too so they don't have a high resistance capacity of all those things …. This community is facing a high problem … in refugee camp was some kind of terrible lack of nutritious food, lack of good, er water … and lack of medical capacities.”

Community member, Melbourne

### Heat is different

Many respondents who were migrants commented that the heat in Adelaide and Melbourne was different to that with which they were familiar. They spoke of the dry heat; that the temperature often does not cool down at night, and that sunburn can be an issue. Moreover, a Sydney community worker said that people in her Asian community were not used to wearing sunscreen as sunburn was rare in their country. A newly arrived community member said he was not aware of the climate in Melbourne before coming to Australia, and compared to Bhutan he found it “*extremely hot, extremely hot*”. Furthermore, it was mentioned by more than one respondent that Australians stereotypically make assumptions about people from hot countries and their ability to cope with the heat:

“The problem we have as Africans in the heat is that the sun here you can actually feel it burning your skin where [as] … sun [in Africa] does not burn your skin.”… “Most Australians think that, especially Africans … are used to heat… But, as I said before, it is a different type of heat…”

Health care worker, Adelaide

### Socioeconomic status, housing and power costs

Narratives revealed that when migrants and refugees arrive they are often unable to gain employment and can face financial disadvantage. Poor educational attainment for some makes this quest more difficult. Low socioeconomic status (SES) can be linked to poor housing, and difficulty in paying utility bills. One respondent also spoke of the sense of “*obligation*” felt by people in his community to send monetary support to family in their home country, adding to financial stress.

Housing was mentioned by most respondents who said that usually rental accommodation for migrants is very basic with no air conditioning and often no fans. Sometimes occupants can stay in these properties as they age and their vulnerability increases. Compared to Adelaide, there were some differences in Melbourne where a lower proportion of homes have air conditioning. A program coordinator said that people once thought they could cope with the heat but now “*because of the changing weather and more hot days, people are installing air-conditioning.”* A manager from Sydney said that central air conditioning should be standard in Australian homes as central heating is in Europe. Another respondent spoke of housing issues for two main groups - older people and new arrivals:

“What we have got is - the two different groups who are impacted, the older people are often in old houses that don’t have insulation and … the houses aren’t good for … the heat. The newly-arrived are in rental properties and often at the lower end of the market too and … don’t necessarily insulate their houses for their tenants.”

Program Coordinator, Sydney

Adelaide has hot summers and the vast majority of homes are air conditioned. In each of the interview sessions in Adelaide, the high cost of power was mentioned as a major barrier to air conditioner usage. This issue was raised to a lesser extent in Sydney, where a notable difference was the numerous clubs and gambling venues offering a cooled, welcoming environment. These are often frequented when the weather is hot, leading to financial stress for gamblers who are then unable to pay their power bills. Rising utility costs are a concern for many including older migrants and low income earners in the general population including those in new and emerging communities:

“…*This is the community in general … the increasing rising costs of electricity is a huge issue and factor…. People still will make that decision consciously not to put their air-conditioner on because they don’t want the stress and the worry about getting that bill.”*

Coordinator, Adelaide

### Language barriers and low literacy

Having poor English proficiency can be a barrier during hot weather, and can increase vulnerability and isolation in people unable to access services, receive information or communicate to others. Language barriers can exist not only for new migrants of non-English speaking backgrounds, but also, as reported by respondents, long-standing elderly migrants who may revert to their first language and culture due to age-related neuro-cognitive conditions. Many older migrants who arrived post World War II, and recent humanitarian entrants, have had minimal if any, schooling and cannot read well even in their native language. These low literacy levels can also affect the transfer of information, the uptake of heat-health messages and the ability to read safety signs (e.g. at the beach), as mentioned by one respondent.

A service manager in Sydney explained that older people in new and emerging communities find it particularly difficult to learn English. Similarly, an Adelaide respondent said this can lead to limited verbal communication as younger family members who were born and raised in refugee camps often do not speak the traditional dialects of their elders. Furthermore, a refugee family in Melbourne said that being unable to understand the language was a “*real barrier*” to being able to access information about extreme heat. Additionally, language barriers can hamper access to health care:

“If I don’t speak English … for example I had someone sick at home - so even if I find a place to help I don’t know how to say it how to describe it, what I need.”

Community Worker, Sydney

### Isolation and transport issues

Respondents spoke of strong social and family connections in CALD communities; however, as mentioned above, certain factors can lead to individuals or families becoming linguistically or socially isolated. In Adelaide, accessing cooler places can be a problem for people without transport options, thereby adding to social isolation and vulnerability during extreme heat. Although asylum seekers, humanitarian entrants and others may lack connections in the community, isolation was mainly spoken about in the context of older people.

“And we do have clients that don’t have English and they are living on their own and some cases they are the only ones in the country. We even have clients who don’t have any other relatives, so that really isolates them.”

Service provider, Adelaide

### Who is vulnerable?

Respondents mentioned that amongst the vulnerable were people from areas in Africa, Bhutan, Middle East and the cool European and Scandinavian countries. Also mentioned were asylum seekers, mothers with babies (particularly single mothers), young children, people with low SES and low income, the homeless, people with poor English and the isolated in CALD communities. People with a disability and their carers, people with mental health problems and multiple chronic illnesses, and those taking certain medications were also vulnerable. Most often mentioned however, were the newly arrived, low SES migrants and refugees in new and emerging communities and who are not acclimatised to the conditions, and older people in migrant communities, especially those who lack English proficiency, as highlighted by these extracts:

“The older ones are particularly vulnerable because of the language and other cultural issues and … - there is an attitude of: we are going to stick it out and cope with it.”

Program Coordinator, Melbourne

“So I would suggest that the newly-arrived because they don’t understand this environment, … they are at a loss about how to cool themselves.”

Program Coordinator, Sydney

There are some similarities in risk factors for these two groups as shown in Table 3 which summarises some of the points previously mentioned.

**Table 3:** Key issues contributing to vulnerability in new arrivals and older migrants

**Issues**	**New arrivals**	**Older, long-term migrants**
Coping with heat	Not used to the dry heat and are unaware of adaptive behaviours	Think they can cope in the heat as they did when they were young
Air conditioning (AC)	Cannot afford power costs associated with AC	A frugal generation, reluctant to use AC
Poor housing	Poor quality rental housing with no AC	Often older, less efficient housing
SES	Low SES, unemployment, financial hardship	Pensioners have low incomes. Can be asset rich, cash poor
Language barrier	Many do not speak English	First language reversion can occur later in life
Low literacy levels	Many are illiterate	Low literacy levels in many older migrants
Many do not drink water	Some not accustomed to drinking tap water	May have incontinence issues
Health conditions	Can have poor mental health, Vitamin D & nutrition deficiencies	Dementia, age-related chronic conditions and disabilities
Clothing	May wear heavy or layered garments	Often over-dress, wear traditional clothes
Isolation	Asylum seekers may lack social connections	Many older persons live in isolation
Transport	Many do not drive and cannot easily get to cooler places	Mobility issues re access to cooler places

Although some respondents stressed the importance of the issue and concerns for their community members in the heat, others thought that in the context of the complexity of issues facing those in the midst of resettlement, weather is unlikely to rank as a priority:

“It comes last for them to know: oh, okay, the sun is burning me or I have to drink water - who cares if I have to drink water or not if I don’t have money to pay my bill, you know, that comes not being essential in a priority.”

Health care worker, Adelaide

## Discussion

This qualitative investigation has given voice to stakeholders and people in cultural and linguistic minorities about the topic of extreme heat in Australia. Whilst the definition of ‘culturally and linguistically diverse’ in Australia is broad, respondents’ narratives related mainly to people or their descendants who have migrated from countries abroad with cultural differences to Australia, and the main spoken language is not English; hence in this instance ‘CALD’ is used as a descriptor in these terms.

This study draws on previous research in Adelaide recognising a need to investigate potential heat-susceptibility in non-Australian born residents [20,21] given the paucity of current literature on this topic [22]. It also builds on international evidence that points to a disparity in the risk of heat-related illness in people of different ethnic/racial backgrounds [10,23-26].

Findings have identified a range of multi-factorial issues that may hamper some migrants and refugees in adapting to periods of very high to extreme summer temperatures in Australia. These relate to cultural factors including wearing garments more suited to cool weather, not drinking enough water, and unfamiliarity with certain aspects of Australian culture including the use of sunscreen. Health issues, socioeconomic disadvantage and poor quality rental accommodation for low income migrants, social isolation, language and literacy barriers limiting access to heat health warning messages, and lack of acclimatisation to the ‘different’ heat in south-eastern Australia can also impact on the potential risk of harm during heat extremes.

The vulnerable individuals in CALD communities were often identified as older people, new arrivals (i.e. who settled in Australia within the last 5 years), and people in new and emerging communities. Older people in general can have declining physical and mental health that can increase heat-susceptibility. However, they generally do not consider themselves to be at risk [27] and are reluctant to using cooling systems [21]. Older people in new and emerging communities may be doubly at risk, particularly if they lack English proficiency skills which can add to isolation and limit access to harm minimisation information. This is mirrored by other studies reporting that ethnic minority language groups can be vulnerable to extreme heat because of exclusion from access to English-based reports and heat information [28,29]. As a consequence there can be a lower uptake of adaptive behaviour messages [23]. Language barriers not only apply to the recently arrived but also the ageing post-war European migrants who can become nostalgic later in life and revert to their primary culture and language, as described by Schmid and Keijzer [30].

Stakeholders mentioned a range of physical and psychological conditions affecting humanitarian entrants and older migrants. In a Sydney study of access to health care for recently arrived refugee families, it was found that few owned a house or car, nearly all were unemployed, and most did not have functional English language skills [31]. There was also the disadvantages of low literacy skills, financial handicap, language barriers, lack of transport, not knowing where to seek help, and poor health knowledge [31]. These findings parallel the narratives of respondents in this study and highlight the barriers for resettled refugees that can hinder acculturation. Not being physiologically and behaviourally acclimatised to the local climatic conditions can influence risk [25,32] and can be a factor in heat-related deaths in Australia [33]. Immigrants of different skin colours and pre-migration climatic experiences commented on the different type of heat in Australia. However, this was not the case in Sydney, where humidity is higher during the summer months [34]. Migration–related factors can influence tolerance and adaptation to extreme heat and it is understandable that newly arrived migrants may suffer in the uniquely dry heat of south-eastern Australia.

Turning on home air conditioners, and using air conditioned cars to drive to cooler places as practiced by most Australian-born families [35] are options unavailable to the financially disadvantaged. This lack of ability to attain thermal comfort during extreme heat has the potential to increase the risk of adverse heat health outcomes. Conversely, using cooling devices is highly protective [36,37], however usage is expensive and we found the high cost of power to be a common barrier mentioned in narratives from Adelaide and Sydney. Adelaide has the third highest household electricity costs in the world behind Denmark and Germany [38], reportedly as a result of the high power demand caused by air conditioner usage during hot weather [39]. Smarter technologies and improvements to housing design are needed to reduce the health impacts of high temperatures [32] and lower the need for home air conditioning. Publicly cooled spaces can be frequented by people not wishing to incur high energy bills at home. Disturbingly however, there was evidence that for some people in new and emerging communities the risk of being marginalised in public can influence adaptive behaviour and was a deterrent to people retreating to shopping centres. This is supported by another study which claims that in a predominantly Caucasian society, “*visibility*” due to different skin colour, attire or accent, can render refugees and others vulnerable to “*street discrimination*” [40].

Notwithstanding these issues migrants, by necessity, can be resilient and have a high adaptive capacity, and certain cultural norms and life experiences can be beneficial to the resettlement process. Enablers to heat adaptation include strong family structure and social networks that exist within collectivist communities. High social capital and having elders live with the family reduces the likelihood of isolation which is known to be linked to societal vulnerability and a risk factor for heat-related mortality [23,41].

Simple harm minimisation behaviours can mitigate the health threat posed by extreme heat, but these are not necessarily intuitive, particularly to those who have not long resided in Australia. Multilingual heat-health advisories could be broadcast via a range of ethnic media outlets and community networks during heatwaves to increase awareness about the health risks of heat exposure including dehydration, and inform about behaviours to minimise the risk of harm in the heat [22]. Furthermore, a better understanding and knowledge of effective health promotion measures within collectivist societies, and the influence of cultural practices and sensitivities on health outcomes, will better inform population health programs and services [42].

This study has several limitations. Sample sizes were relatively small and there were few interviews at the community level. However, this scoping study has laid the foundations for a further study currently being undertaken involving community members. The migrant population of Australia is vastly heterogeneous and findings are not intended to be generalisable beyond the scope of the study. Findings may reflect problems that exist in only a minority of migrants and refugees if recruitment inadvertently resulted in a biased sample. Indeed, among immigrants arriving as part of the skilled migration program employment rates can be low and English proficiency high [43] and it would therefore be less likely that heat risks in this group would differ to that of the equivalent Australian-born population. Nevertheless, this study has given voice to those who have expressed genuine concerns about the potential impact of extreme heat on the disadvantaged with cultural and linguistic vulnerabilities, and an unmet need for access to appropriate information about adaptive behaviours. Further qualitative and quantitative research is required to investigate potential disparities in the impacts of extreme heat on minority groups in Australia.

## Conclusions

Stakeholders within and working with CALD communities have observed sociocultural barriers that can hinder effective adaptation of migrants to extreme heat in Australia. Low income, recently arrived non-English speaking migrants, as well as isolated and older migrants who lack access to a cooled environment are of particular concern. With migration increasing, first generation migrants becoming part of the ageing population, and climate change bringing more frequent and intense periods of extreme heat, policymakers need to be mindful of the need for culturally and linguistically competent strategies for disseminating risk messages and heatwave warnings.

## Competing interests

The authors declare that they have no competing interests.

## Authors’ contributions

AH was responsible for data collection, data analysis and drafting the manuscript. AS participated in data collection. PB, MN, AS, JB, YT, VS, LW, G-SH and LM were members of the Research Reference Group, providing intellectual guidance in the design of the study and assisting in recruitment across the three study sites. All authors read and approved the final manuscript.

## Authors’ information

Alana Hansen is the Submitting author.

## Pre-publication history

The pre-publication history for this paper can be accessed here:

http://www.biomedcentral.com/1471-2458/14/550/prepub

## Supplementary Material

Additional file 1 Qualitative research review guidelines – RATS checklist. Click here for file
