# Supplementary material for: Extreme heat and cultural and linguistic minorities in Australia: perceptions of stakeholders
Source: BMC Public Health. 2014 Jun 3;14:550. doi: 10.1186/1471-2458-14-550 (PMC4107973; doi:10.1186/1471-2458-14-550)
Supplement: Additional file 1 — Qualitative research review guidelines – RATS checklist. [file 1471-2458-14-550-S1.docx]

**Qualitative research review guidelines – RATS checklist**

| **ASK THIS OF THE MANUSCRIPT** | **THIS SHOULD BE INCLUDED IN THE MANUSCRIPT** | **AUTHORS’ RESPONSE** |  |
| --- | --- | --- | --- |
| **R Relevance of study question** |  |  |  |
| Is the research question interesting?  Is the research question relevant to clinical practice, public health, or policy? | Research question explicitly stated  Research question justified and linked to the existing knowledge base (empirical research, theory, policy | We believe the research question is interesting and very relevant to public health policy and practice.  The research question, stated in the aim of the research in the last sentence of the Background, is justified and linked to the existing knowledge base. |  |
| **A Appropriateness of qualitative method** |  |  |  |
| Is qualitative methodology the best approach for the study aims?   - *Interviews:* experience, perceptions, behaviour, practice, process - *Focus groups:* group dynamics, convenience, non-sensitive topics - *Ethnography:* culture, organizational behaviour, interaction - *Textual analysis:* documents, art, representations, conversations | Study design described and justified i.e., why was a particular method (e.g., interviews) chosen? | Qualitative methodology, using interviews and focus groups was, we feel, the best approach to address the research question. The diversity of CALD groups in Australia meant that community-specific issues needed to be explored which was more appropriate using interviews rather than eg a standardised survey  The study design has been well described and justified in the Methods section. |  |
| **T Transparency of procedures**  *Sampling* |  |  |  |
| Are the participants selected the most appropriate to provide access to the type of knowledge sought by the study?  Is the sampling strategy appropriate? | Criteria for selecting the study sample justified and explained   - *theoretical:* based on preconceived or emergent theory - *purposive:* diversity of opinion - *volunteer:* feasibility, hard-to-reach groups | In the 2nd paragraph of the Methods section, we have justified why the study sample of stakeholders was selected by purposive methods and how snowball sampling resulted in the recruitment of volunteer respondents from two CALD communities. We believe this sampling strategy was appropriate to address the research question. |  |
| *Recruitment* |  |  |  |
| Was recruitment conducted using appropriate methods? | Details of how recruitment was conducted and by whom | Details of how recruitment was conducted can be found in the Methods section. Potential respondents were contacted by telephone and/or email and interested persons followed up. |  |
| Is the sampling strategy appropriate? |  | We feel this was an appropriate method of recruitment and provided stakeholders with the opportunity to decline to be part of the study. |  |
| Could there be selection bias? | Details of who chose not to participate and why | It is acknowledged that in any study involving recruitment there could be selection bias if the participants are only those who deem the research topic to be an issue of concern. However, as this is a growing public health concern generally, respondents working in local government, health and the various communities were likely to reflect more widespread concerns. Non-responders may have different views. We have stated in the Discussion that “Findings may reflect problems that exist in only a minority of migrants and refugees if recruitment inadvertently resulted in a biased sample”. In reference to those who chose not to participate, on page 8 we have added the sentence: “On occasions the primary contact declined to participate and suggested a secondary contact more experienced in the research topic.” |  |
| *Data collection* |  |  |  |
| Was collection of data systematic and comprehensive? | Method(s) outlined and examples given (e.g., interview questions) | The methods are clearly outlined and the interview questions are referred to in the first paragraph on page 9. Reference 15 (Hansen et al 2013) containing the full list of questions, is cited. |  |
| Are characteristics of the study group and setting clear? | Study group and setting clearly described | The study group and setting are clearly described |  |
| Why and when was data collection stopped, and is this reasonable? | End of data collection justified and described | As the research topic related to extreme heat, data collection took place “in the warm months between December 2011 and April 2012” (page 8). Conducting interviews in the winter may have resulted in recall bias. |  |
| *Role of researchers* |  |  |  |
| Is the researcher(s) appropriate? How might they bias (good and bad) the conduct of the study and results? | Do the researchers occupy dual roles (clinician and researcher)? Are the ethics of this discussed? Do the researcher(s) critically examine their own influence on the formulation of the research question, data collection, and interpretation? | The researcher does not occupy dual roles with conflicting interests, and is impartial in the research process. The research question arose as a recognised gap in knowledge, from previous studies undertaken by colleagues and a review of international literature. Members of the Research Reference Group (mentioned on page 8 and 9) guided the research process and assisted in focus groups and interpretation of data. |  |
| *Ethics* |  |  |  |
| Was informed consent sought and granted? | Informed consent process explicitly and clearly detailed | Informed consent was sought and granted from each respondent. In the Methods we state “Informed, written consent was provided by respondents prior to the commencement of the interviews. For one community group information sheets and consent forms were translated…” |  |
| Were participants’ anonymity and confidentiality ensured? | Anonymity and confidentiality discussed | Anonymity and confidentiality was discussed in the information sheets provided to each respondent. “..*and confidentiality was assured”* has been appended to the last sentence on page 8. |  |
| Was approval from an appropriate ethics committee received? | Ethics approval cited | Ethics approval was granted from multiple ethics committees as mentioned at the end of the Methods section |  |
| **S Soundness of interpretive approach**  *Analysis* |  |  |  |
| Is the type of analysis appropriate for the type of study?   - *thematic:* exploratory, descriptive, hypothesis generating - *framework:* e.g., policy - *constant comparison/grounded theory:* theory generating, analytical   Are the interpretations clearly presented and adequately supported by the evidence? | Analytic approach described in depth and justified  *Indicators of quality:* Description of how themes were derived from the data (inductive or deductive)  Evidence of alternative explanations being sought  Analysis and presentation of negative or deviant cases | We believe our analytic approach (thematic analysis and the framework approach) and how themes were derived, have been well described in the Data Analysis section. The framework approach is particularly relevant for applied policy research.  We have stated that our “approach enhances rigour, transparency and validity of the analytic process [[18](#_ENREF_18)]. Analysis was both deductive, with categories derived from prior knowledge, and inductive, with categories emerging purely from the data [[19](#_ENREF_19)].”  Our coding process is transparent and quality assured as Table 2 presents evidence of data and their allocated themes. |  |
| Are quotes used and are these appropriate and effective? | Description of the basis on which quotes were chosen  Semi-quantification when appropriate  Illumination of context and/or meaning, richly detailed | Several illustrative quotes have been used as evidence of particular points made in the text. We believe these have been used appropriately to effectively demonstrate how conclusions have been drawn from the narratives. |  |
| Was trustworthiness/reliability of the data and interpretations checked? | Method of reliability check described and justified e.g., was an audit trail, triangulation, or member checking employed? Did an independent analyst review data and contest themes? How were disagreements resolved? | On the Research Reference Group were four experienced qualitative researchers, one of whom checked the interpretation of the analysis undertaken by the researcher. |  |
| *Discussion and presentation* |  |  |  |
| Are findings sufficiently grounded in a theoretical or conceptual framework?  Is adequate account taken of previous knowledge and how the findings add? | Findings presented with reference to existing theoretical and empirical literature, and how they contribute | Thematic analysis was undertaken to identify themes relevant to the research question. The findings are presented in the context of existing published research in the international literature. This paper contributes an Australian perspective, which to date, has been a gap in knowledge. |  |
| Are the limitations thoughtfully considered? | Strengths and limitations explicitly described and discussed | The limitations of the study are clearly outlined in the last paragraph of the Discussion |  |
| Is the manuscript well written and accessible? | Evidence of following guidelines (format, word count)  Detail of methods or additional quotes contained in appendix  Written for a health sciences audience | We have followed the journal’s guidelines for authors and written for a health sciences audience. We have not used an appendix. |  |
| Are red flags present? These are common features of ill-conceived or poorly executed qualitative studies, are a cause for concern, and must be viewed critically. They might be fatal flaws, or they may result from lack of detail or clarity. | *Grounded theory:* not a simple content analysis but a complex, sociological, theory generating approach  *Jargon:* descriptions that are trite, pat or jargon filled should be viewed sceptically  *Over interpretation:* interpretation must be grounded in "accounts" and semi-quantified if possible or appropriate  *Seems anecdotal, self evident:* may be a superficial analysis, not rooted in conceptual framework or linked to previous knowledge, and lacking depth  *Consent process thinly discussed:* may not have met ethics requirements  *Doctor-researcher:* consider the ethical implications for patients and the bias in data collection and interpretation | *Grounded theory:* we used thematic analysis of the narratives and a framework approach. We do not claim to have used grounded theory as a theoretical basis to our work.  *Jargon:* our descriptions are thoughtfully constructed and evidence-based. The use of jargon has been avoided.  *Over interpretation:* we have been transparent in our analysis to enhance rigour. Our interpretation of key points is supported by the use of quotes and a comprehensive table of themes.  *Seems anecdotal, self evident:* we believe our analysis, although not rooted in a conceptual framework, has been undertaken with scientific rigour. Findings are linked to evidence and previous knowledge stemming from the international and Australian literature.  *Consent process thinly discussed:* we have met the ethics requirements of 3 institutions and our consent process has been discussed.  *Doctor-researcher:* our respondents were not patients and therefore there was no ethical doctor-researcher implications in terms of data collection and interpretation. |  |
